# Reducing Cost of Rabies Post Exposure Prophylaxis: Experience of a Tertiary Care Hospital in Pakistan

**DOI:** 10.1371/journal.pntd.0004448

**Published:** 2016-02-26

**Authors:** Naseem Salahuddin, M. Aftab Gohar, Naila Baig-Ansari

**Affiliations:** 1 Infectious Disease Division, Department of Medicine, The Indus Hospital, Karachi, Pakistan; 2 Emergency Department, The Indus Hospital, Karachi, Pakistan; 3 Indus Hospital Research Center, The Indus Hospital, Karachi, Pakistan; American Heart Association, UNITED STATES

## Abstract

**Background:**

Rabies is a uniformly fatal disease, but preventable by timely and correct use of post exposure prophylaxis (PEP). Unfortunately, many health care facilities in Pakistan do not carry modern life-saving vaccines and rabies immunoglobulin (RIG), assuming them to be prohibitively expensive and unsafe. Consequently, Emergency Department (ED) health care professionals remain untrained in its application and refer patients out to other hospitals. The conventional Essen regimen requires five vials of cell culture vaccine (CCV) per patient, whereas Thai Red Cross intradermal (TRC-id) regimen requires only one vial per patient, and gives equal seroconversion as compared with Essen regimen.

**Methodology/Principal Findings:**

This study documents the cost savings in using the Thai Red Cross intradermal regimen with cell culture vaccine instead of the customary 5-dose Essen intramuscular regimen for eligible bite victims. All patients presenting to the Indus Hospital ED between July 2013 to June 2014 with animal bites received WHO recommended PEP. WHO Category 2 bites received intradermal vaccine alone, while Category 3 victims received vaccine plus wound infiltration with Equine RIG. Patients were counseled, and subsequent doses of the vaccine administered on days 3, 7 and 28. Throughput of cases, consumption utilization of vaccine and ERIG and the cost per patient were recorded.

**Conclusions/Significance:**

Government hospitals in Pakistan are generally underfinanced and cannot afford treatment of the enormous burden of dog bite victims. Hence, patients are either not treated at all, or asked to purchase their own vaccine, which most cannot afford, resulting in neglect and high incidence of rabies deaths. TRC-id regimen reduced the cost of vaccine to 1/5^th^ of Essen regimen and is strongly recommended for institutions with large throughput. Training ED staff would save lives through a safe, effective and affordable technique.

## Author Summary

Rabies is a killer disease caused by the rabies virus that is present in the saliva of rabid animals, mainly the dog. Once symptoms become apparent, death is inevitable. However, rabies can be prevented if correct post exposure prophylaxis (PEP) is instituted as soon as possible after a dog bite and before symptoms of rabies begin. In Pakistan, government hospitals treat 50–70 new bite victims each day. Many still dispense the free but poor quality nerve tissue vaccine that is often ineffective and fraught with serious adverse reactions. Hospital administrators consider PEP too expensive to be administered free of cost. The Indus Hospital (TIH), Karachi is a private teaching hospital which provides free medical care to all. From July 2013-June 2014, 2,983 new bites were seen in the ED, and rather than use the Essen regimen of five full vial intramuscular doses per patient over 28 days, we administered the WHO-approved Thai Red Cross-intradermal (TRC-id) 4-dose regimen. The use of the TRC-id regimen resulted in 80% cost savings over the Essen regimen. In resource-poor settings, we advocate training of ED personnel in TRC-id regimen, which, ultimately, will result in less vaccine consumption, more patient compliance and complete treatment, resulting in more lives being saved.

## Introduction

Rabies is a grave zoonotic infection transmitted to humans or animals by the bite of a rabid animal, usually a dog. Dog rabies is almost non-existent in Europe, North America and Australia, but it mainly exists in the poorer strata of society in the developing world. Animal and human rabies is prevalent in both urban and rural areas virtually all over Pakistan. Rabies is invariably fatal, yet completely preventable if post exposure prophylaxis (PEP) is applied in a timely and correct manner. WHO has prepared standard recommendations for PEP, centered upon circumstances of the biting animal and wound severity. It strongly recommends immediate and thorough wound washing with soap and water, followed by vaccine and infiltration of Rabies immune globulin (RIG) in severe bites[1].

Unfortunately, awareness of rabies prevention in most developing countries of the world, and especially in Pakistan, is insufficient. Most victims do not report to a health center[2,3]; nor do they wash the bite wound with soap and water. Instead, they apply home remedies. Even in some large health care centers, the wound severity is not assessed correctly, and hence appropriate decisions about usage of vaccines and RIG are not made, which, in the worst case, causes rabies deaths[2,4]. Moreover, Pakistan and Myanmar are the only two countries in the world where the obsolete nerve tissue vaccine is still produced and provided to government hospitals and clinics. In one study from Pakistan, the nerve tissue vaccine was found to have zero potency[5]. Cell or tissue culture vaccines have long replaced nerve tissue vaccine in almost all countries of the world, as they are proven to be safe and effective. Several imported culture vaccines are available in Pakistan[6–9], yet, many institutions do not practice correct PEP either because the staff are not trained, or vaccine and RIG are not provided because they are considered “unsafe” or “unaffordable.”[10]

Since 1984 two regimens for intramuscular delivery, approved by WHO, have been in practice. However, for low income persons, the cost of the 4 or 5-dose regimen could cost their full month’s salary; hence they either forego PEP or approach government-run hospitals for treatment, which also cannot bear the cost for the large number of patients seen on any given day.

In 2005,WHO Expert Consultation on Rabies approved the low dose modified Thai Red Cross intradermal (TRC ID) 4-dose regimen as being safe, immunogenic and economical. Several studies have demonstrated its safety and immunogenicity. TRC ID regimen elicits equivalent immune response as the 5 dose intramuscular Essen or 4-dose Zagreb regimens[11–13]. TRC ID requires 0.1 ml per injection, whereas IM regimen requires 0.5 ml or 1.0 ml of the same vaccine. This practice is used in most Asian countries, and because of cost reduction, it has been instrumental in expanding delivery service so that more victims now receive PEP, and in these case rabies is averted[13,14].

The Indus Hospital Karachi (TIH) is a 150-bed free tertiary care hospital in Pakistan’s largest city Karachi. The catchment area of the hospital is approximately 2.5 million persons primarily of low socio-economic status. The Rabies Prevention Center (RPC) was established in the Emergency Department (ED) of the hospital in 2008.The center policy is to use the 4-dose TRC-id regimen for vaccination of all WHO category II and III wounds, whereas equine rabies immunoglobulin(ERIG) is injected in WHO category III wounds in addition to the intradermal vaccine according to guidelines.[1,15]

Our primary objective is to describe the RPC’s throughput of rabies-prone bites between 2009 and 2014 as well as to demonstrate the cost effectiveness of TRC-id as compared with the Essen five-dose intramuscular regimen in a one-year period.

## Materials and Methods

This is a descriptive study of all victims of rabies- prone animal bites reported to the RPC of TIH between January 2009 and December 2014. Information on all patients who presented to the RPC with rabies prone bite was collected on a pre-coded form by the RPC coordinator. Age, gender, the time and type of incident, site and categorization of wounds on the basis of WHO classification were documented along with a profile and circumstances of the offending animal, and its behavior as reported by the victim and/or attendant. First aid and medical care provided in the RPC was documented, as well as the type and route of administration of vaccine and infiltration of RIG in category III wounds. Patients presenting with rabies were also documented. Since there is no provision in Karachi for quarantining or testing animal brain tissue for rabies in animals, all bites were assumed to be rabies prone, and all bite victims received PEP.

Each patient was interviewed about circumstances of the animal bite, condition of the animal, and assessment of the number and depth of the victim’s wound. All patients received wound cleansing for at least fifteen minutes with soap and flowing water, followed by application of chlorhexidine antiseptic. All WHO category 2 wounds were treated either with WHO pre-qualified Purified Chick Embryo Cell Vaccine (PCECV 1.0 ml vial) or Purified Verocell Rabies Vaccine (PVRV 0.5 ml vial), both of which have proven safety and immunogenicity, and are approved for intradermal injection. Since PCEC is dispensed in 1.0 ml, one vial was shared among five persons; whereas PVRV is dispensed in 0.5 ml, hence one vial was shared between 2.5 persons. Since a reconstituted vial, once opened, should not be stored for more than six hours, the left- over vaccine was often wasted.

Human RIG is prohibitively expensive and is not used in our RPC. Equine RIG (ERIG) was previously known to be associated with hypersensitivity reactions, but current productions of ERIG are highly purified and virtually free of serious reactions. ERIG, calculated for 40 IU/kg body weight was infiltrated into the wound/s as much as anatomically possible, and the remaining injected into a muscle away from the vaccine site, and the patient observed for at least an hour after injection.[16] Each patient or his/her attendant was counseled about the importance of completing the vaccine series, and to return on days 3, 7 and 28 for the remaining injections. An appointment card with the schedule was given.

TIH pharmacy dispenses vaccine and ERIG to the RPC. Audit of vaccine and ERIG consumption were obtained for July 2013 through June 2014.The total number of patients treated, and their follow through for completion of doses were recorded. For cost effectiveness, calculations, record of vaccine and ERIG consumption, and cost of these biological were obtained through pharmacy and the finance departments for the same period.

## Results

**Fig 1:**
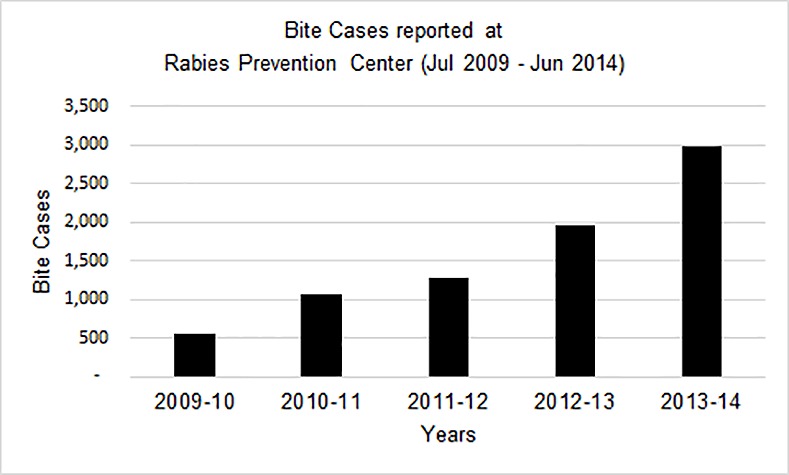
Bite cases throughput at Rabies Prevention Center (2009–2014).

In the past six years, 9,507 victims of animal bite were registered at the RPC. Majority were males (87%) and mean age of victims was 25 years (± 16.3 SD). The numbers treated at the facility increased progressively each year as TIH’s reputation for good quality and free medical care became widespread. (Fig 1)

Profiles of 2983 bite patients seen between July 2013- June 2014 were analyzed (Table 1); of which 67 did not require PEP as they had no-risk bites. Nearly 97% were Category II and III bites, and required PEP. Of those requiring vaccine, 2188 (73%) completed the 4-dose vaccine series. A few bite victims (n = 117) were advised to skip the third dose if the animal was reliably reported to be alive and healthy. The fourth dose on day 28 was injected, converting post exposure to pre exposure prophylaxis (PrEP). In case of a bite sometime in the future, only two booster doses would be required, ie one dose of vaccine intramuscularly or intradermally at one site on both days 0 and 3. Rabies immunoglobulin is not indicated. Vaccination was not advised for 38 patients for various reasons (dog was reliably vaccinated, or the patient visited more than 10 days after a bite and the animal was alive and healthy). After one or two doses, 573 patients either defaulted or were inadvertently not recorded. Additionally, patients were excluded from ERIG if they had received a vaccine within a week before presenting to our facility, since it is not recommended to administer RIG as it blunts antibody response.

**Table 1:** Profile of bite victim throughput seen at The Indus Hospital Rabies Prevention Center (July 2013-June 2014).

	No of patients	%
	n = 2983	
Gender		
Male	2604	87.3%
Female	379	12.7%
Age, years		
0–5	197	6.6%
6–17	1248	41.8%
≥18	1538	51.6%
WHO Category		
Cat I	67	2.3%
Cat II	1528	51.2%
Cat III	1388	46.5%
Vaccination Completed	2188	73.3%
Pre Exposure Prophylaxis	117	4.0%
Incomplete vaccination	573	19.2%
PEP not required	105	3.5%

1388 (46.5%) dog bite victims presented with Category III wounds, of whom 1108 required RIG. 280 were excluded, based on our exclusion criteria, ie. the biting animal was reliably vaccinated, the victim had received vaccine more than a week before presenting to the RPC, or the animal was known to be alive 10 days or m ore after the bite. We did not encounter any serious reaction with ERIG. The cost of a 5 ml vial of ERIG containing 1000 IU is around PKR 1,000 (USD 10). The dose is calculated per weight of the individual. Hence, a small child required 0.5 to 1 vial, and an adult 2–3 vials. The number of vials of ERIG consumed for 1108 patients was 1261 vials, and cost PKR 1,261,000 (USD 12,610). The number of vaccine vials consumed to treat 2300 patients with TRC id regimen was 2400. (Five patients brought their own vials for IM injection). A vial of cell culture vaccine costs PKR 547. The total cost was PKR 1,312,800 (USD 13,128). The sum of treating 2300 patients with proper PEP (2300 required vaccine alone or vaccine plus ERIG) was PKR 2,573,800 (USD 25,738) or PKR 1,200 (USD 12.00) per patient treated. (Table 2)

**Table 2:** Consumption and cost of biologicals for Rabies PEP July 2013 to June 2014 at TIH.

Biological	N of patients	# of vials consumed	Avg # of vials/patient	Total cost of vials	Cost /patient
Vaccine (for Cat II + Cat III)	2300	1800 PCEC 1 ml + 600 PVRV 0.5 ml	1.04	PKR 1,312,800 (USD 13,128)	PKR 570.8 (USD 5.70)
ERIG (for Cat III only)	1108	1261	1.138	PKR 1,26,1000 (USD 12,610).	PKR 1138.0 (USD 11.38)
Vaccine + ERIG				PKR 2,573,800 (USD 2,557,38)	PKR 1200 (USD 12)

Abbrev: PCEC Purified Chick Embryo Cell; PVRV Purified Verocell Rabies Vaccine; ERIG Equine rabies immunoglobulin; PKR Pakistan Rupee; USD US Dollar

If Essen regimen using 5 vials per patient had been used, the cost for the same number of patients treated would have been PKR 6,290,500 (USD 62,905) (Table 3). The total amount of money saved for vaccine was PKR 4,977,700 (USD 49,777).

**Table 3:** Cost savings of vaccine by using TRC–id regimen for 2300 patients.

Regimen	Visit Schedule (# of visits)	# of vials consumed	Cost per patient	Total Cost	Cost Saving
Essen IM	0,3,7,14,28 (5)	11,500	PKR 2735 (USD 27.35)	PKR. 6,290,500 (USD 62,905)	
TRC ID (PEP)	0,3,7,28 (4)	2400	PKR 570.8 (USD 5.70)	PKR 1,312,800 (USD 13,128)	PKR 4,977,700 (USD 49,777)

Throughout the six-year period we have encountered only two cases of rabies among patients who received PEP at our hospital; both had not returned to complete the vaccine course because of long distances from the hospital.

## Discussion

WHO-recommended protocol for PEP includes prompt wound washing, vaccine for Category 2 and 3 bites, and infiltration of RIG into and around the wound in Category 3 bite[1,17]. A survey of HCWs in most EDs in Pakistan revealed that they understood and practiced the Essen Regimen, and would like to infiltrate RIG but were fearful of side effects and its high cost. They were aware of HRIG but found it too costly; they knew about ERIG but believed it caused hypersensitivity reaction frequently and were afraid to use it. Hence, they had never used RIG as it was not purchased in their institution[10]. Therefore they inject cell culture vaccine IM for 1 or 2 doses provided through their center, and ask the patient to purchase the remaining doses. The study showed that RIG was rarely infiltrated, unless the wound was on the head or neck[10]. The cost of travel to the center and loss of time from work are also to be accounted for; hence patients frequently renege on completing the full course. Failure of correct or incomplete PEP is responsible for rabies, which is irreversible and always fatal. Other centers, too have expressed difficulties in managing severe and unusual bites, administering immunoglobulin, logistics of travel and financial constraints, all of which are liable for high failure rate of PEP[18].

The decision to provide rabies PEP frequently presents a dilemma, especially if the circumstances of the biting animal are unclear. In many situations rabies PEP is given to persons whose risk of exposure to the virus is very low or none, raising the cost of care. However, justification is sought from the fact that since rabies is a fatal disease, one cannot “take a chance”. In an ideal situation, the biting animal should be quarantined and kept under observation and tested to rule out rabies[19]. Thus, many bite victims would be saved from receiving PEP, and the cost of precious RIG and vaccine would be saved. Unfortunately, there is no provision in the region for isolating suspected dogs or for diagnosing animal rabies.

Issues that remain unaddressed pertain to the control of animal rabies. Veterinarians advocate mass dog vaccination campaigns to improve herd immunity and reduce the burden of human rabies[20]. Animal rabies control requires more attention, and must be done through local municipalities. Rabies elimination programs focused mainly on mass vaccination of dogs are largely justified by the future savings of human rabies prevention programs.(20)

Our study supports two essentials: firstly, both ERIG and TRC ID regimen with cell culture vaccine are the essential standard of care and are safe and effective; secondly, the cost of vaccine with or without ERIG is affordable for any institution giving free or subsidized care. The caveat is that at least two bite victims should present within 6–8 hours, after which the vaccine must be discarded, and is wasted. Our experience shows that once the hospital becomes recognized for its commitment to care of animal bite patients, the number of ED visits rise and the cost of PEP actually decreases. Thus, more lives are saved from a cruel death due to rabies.

### Conclusion

This study should dispel misgivings among patients and health care givers that management of dog bites is problematic and fraught with danger, and that it is prohibitively expensive. We have shown that in a large Rabies Prevention Center situated in an ED, using TRC-id regimen with quality cell culture vaccine, plus ERIG in all deep wounds is manageable as well as cost effective. We strongly recommend that HCWs obtain training in WHO recommended PEP, and that only high quality and tested cell culture vaccine and ERIG be made available in all EDs. The 5-dose IM Essen regimen may be reserved for smaller settings where only an occasional patient presents. Experience and good clinical judgment are essential in preventing human rabies.

### Definitions

#### Clinic throughput

The number of bite patients presenting to a clinic for the first time in need of PEP.

#### Vial size

Purified Cell Embryo Culture Vaccine (PCECV) 1 ml, can be divided between 5 persons. Purified Verocell Rabies Vaccine (PVRV) 0.5 ml, can be divided among 2.5 persons

#### PEP not required

Category 1 bite, or bite from healthy vaccinated animal

#### Vaccine wastage

Opened vials must be refrigerated. Unused reconstituted vaccine must be discarded after 8 hours. Occasionally, if a skin bleb is not raised, the vaccine musts be injected again

#### Patient compliance

Patients coming from far off places frequently do not return for course completion, thus placing themselves at risk of rabies.

#### Vaccination completed

All 4 doses received

#### Pre Exposure Prophylaxis (PreP)

If the dog is alive and healthy at Day 7, the third dose is eliminated and the fourth dose given on Day 28 and converted to PreP. Thus, there is a saving of 1 dose (0.2 ml) vaccine per patient.
